# Patterns of Agricultural Crop Damage by Wild Boar (*Sus scrofa*) in South-Western Poland

**DOI:** 10.3390/ani15233500

**Published:** 2025-12-04

**Authors:** Bogusław Bobek, Anna Chrzan, Jakub Furtek, Małgorzata Kłyś, Dorota Merta, Marta Wojciuch-Płoskonka

**Affiliations:** 1 Institute of Biology and Earth Sciences, University of the National Education Commission, Podchorążych 2, 30-084 Kraków, Poland; anna.chrzan@uken.krakow.pl (A.C.); malgorzata.klys@uken.krakow.pl (M.K.); dorota.merta@uken.krakow.pl (D.M.); 2 Polish Wildlife Foundation, Żołnierska 31, 30-735 Kraków, Poland; jakubfurtek@gmail.com (J.F.); martawojciuch@op.pl (M.W.-P.)

**Keywords:** crop damage, conflict mitigation, farmlands, hunting bag, seasonal changes

## Abstract

Studies on agricultural crop damage inflicted by wild boar (*Sus scrofa*) were conducted in hunting districts located in Lower Silesia, south-western Poland. The statistical analysis of damaged agricultural crops was based on documentation obtained via survey from hunting associations managing 81 hunting districts. For each hunting district (mean area 43.1 km^2^), this documentation detailed the area of damaged crops (maize, various cereals, root crops, rapeseed, grasslands, and other crops), the date of the damage, and the value of compensation paid to farmers. During three consecutive hunting seasons (2013/14–2015/16) the area of damaged farmlands was amounted to 2098.2 hectares. Maize constitutes 43.9% of the total damage area, while for various cereal crops and grasslands, the figures were 29.5% and 13.2%, respectively. The mean damage compensation per hectare amounted to €421.0, the highest being for root crops (€942.8/ha) and the lowest for grasslands (€214.8/ha). A positive correlation was shown between the wild boar harvest rate and the percentage of farmland area damaged by these animals. Between the 2015/16 and 2022/23 hunting seasons, a decline in the density of harvested wild boar from 1.99 to 1.05 individuals reduced the area of damage from 0.335 ha/km^2^ to 0.164 ha/km^2^ of farmland. Population density control has been suggested as the most effective method of protecting agricultural crops against wild boar.

## Simple Summary

Research on agricultural crop damage inflicted by wild boar was conducted in hunting districts located in Lower Silesia, south-western Poland. In Poland, hunting associations are obligated to pay farmers compensation for damage caused by wild boar to agricultural crops and to maintain detailed documentation regarding the damage area, date of occurrence, and compensation amount. These data, along with wild boar hunting bag (harvest) figures, were obtained from hunting associations via survey for three consecutive hunting seasons (2013/2014–2015/2016) and for the 2022/2023 hunting season. The data obtained also included the farmland and forest area of each hunting district. Wild boar caused damage to 15 different agricultural crops. For statistical analysis, crop damage results were grouped into grasslands (meadows and pastures), maize, various cereal crops (barley, wheat, oat, rye), root crops (potato, sugar beets, carrot), rapeseed, and “other crops” (clover, lupine, strawberry, vetch). The percentage share of the area of the six damage categories in the total area of damaged crops, and the overall amount of compensation paid to farmers, were separately presented for spring, summer, autumn, and winter. Statistical analysis demonstrated a significant positive correlation between the wild boar harvest rate, used as a population density proxy, and the percentage area of damaged farmlands.

## 1. Introduction

In Europe, during recent decades, there has been a rapid increase in the abundance and range of wild boar (*Sus scrofa*) [1,2,3,4]. This is evidenced by the number of culled wild boar, which increased from 865 thousand to approximately 2.2 million between 1982 and 2013 [2], and reached a value of 3.3 million individuals in 2025 [5]. This trend was driven by an increased reproductive rate of wild boar [6,7] due to the expansion of cereal, and especially maize, acreage during the period discussed, which provided them with high-energy food resources [8]. Additionally, the quantity of high-quality forage for wild boar was also influenced by the increased frequency of heavy mast years of oaks (*Quercus* sp.) and beeches (*Fagus sylvatica*) [9]. Mild winters decreased wild boar mortality levels and enabled the colonization of Northern European territories by these animals [10]. The increase in wild boar abundance was also caused by a lack of knowledge among hunters regarding population size and demographic parameters. As a result, the magnitude of the hunting bag was lower than the net population increase [2,11,12].

In Europe, the current wild boar abundance, in addition to the aforementioned habitat, climatic, and hunting bag variables, is also affected by the occurrence of the African swine fever virus [13]. However, the lack of reliable data on wild boar abundance means that the size of the hunting bag for this species is treated as a proxy for the relative abundance or population density of the studied populations. Data from 2023 and 2024 indicate that among 31 European countries with a total area of 5,575,806 km^2^, the average annual wild boar culling was 0.597 individuals per km^2^, ranging from 0.00004 individuals per km^2^ in Norway to 3.27 individuals per km^2^ in the Czech Republic. The annual wild boar hunting bag was the highest in France and the lowest in Norway, amounting to 863 thousand and 140 individuals, respectively. In Poland, 174 thousand wild boar were culled in the 2023/2024 hunting season, equating to 0.56 individuals per km^2^ of the country’s area (FACE 2025) [5].

High densities of wild boar are the cause of many socio-economic and ecological conflicts with humans [14,15,16,17,18,19,20,21]. However, in many European countries, the most important of the conflicts concerns the damage done by this species to agricultural crops [22,23,24,25,26,27,28] and pig industry animals as a result of African swine fever transmission from free-roaming wild boar populations [29,30,31,32,33].

The hunting season for wild boar (*Sus scrofa*) in Poland spans from March to February of the subsequent year. While individual hunts are permitted throughout the year, collective hunts utilizing drivers and dogs are typically organized between October and January.

Each hunting club operates as a small commercial business, required to maintain a full ledger detailing all financial transactions related to game management. This expense documentation includes specific data on the areas, dates, and species composition of agricultural crops damaged by wild boar, alongside records of the compensation sums paid, the number of damage cases, and the names of affected farmers. These comprehensive data were leveraged in this study to determine the area and species composition of damaged agricultural crops, as well as the damage compensation figures, within a diverse forest-farmland environment in south-western Poland.

The procedure for reporting and estimating damage caused by wild boar to agricultural crops is formally regulated by the Ministry of the Environment [34,35,36]. These regulations stipulate that hunting clubs leasing hunting districts are responsible for providing compensation to farmers for losses incurred on farmlands. However, in cases where a hunting district is managed by the state forest, the forest district assumes responsibility for paying the damage compensation. The Polish damage assessment methodology utilizes a hybrid system, combining the measurement of the damaged surface area (spatial extent) with a biometric sampling method (field plots) to determine the percentage of destruction (intensity). Compensation is calculated based on the reduced area, defined as the product of the damaged surface and the percentage of destruction [37].

The damage registration and compensation process is structured in three distinct stages. First, the aggrieved farmer must report the damage to the local state forest service within 3 days of its discovery. Second, within 7 days of this report, the damaged cultivated crop areas are assessed by a three-person committee comprising representatives from the local county office, the hunting club, and the affected landowner. This on-site inspection includes taking measurements of the precise area of agricultural crops damaged by wild boar [37]. In the third and final step, data from the Central Statistical Office (GUS) [38] are utilized to calculate the potential yield of the specific agricultural species. The compensation ultimately paid to the farmer is equivalent to the potential yield of the given crop multiplied by its monetary value in the local market.

However, this system does not ensure unconditional compensation. The farmer forfeits the right to indemnification if agrotechnical errors are identified, if the crop is not harvested within the regulatory period (14 days post-harvest), if consent to hunt on the land is withheld, or if cooperation with the hunting association on preventive measures (e.g., erecting protective devices) is refused. Consequently, the lack of active farmer participation in crop protection is de facto considered equivalent to forfeiting the right to compensation.

It is noteworthy that the use of measures such as diversionary feeding, fencing, gustatory and odor repellents, various acoustic scaring devices, and optical deterrents does not significantly affect the percentage of agricultural crop area damaged by wild boar [22,23,24,25,26,27,28]. Conversely, this damage metric tends to decrease following a reduction in the wild boar relative population density, an increase in the proportion of forest area within the farmland-forest environment, and the occurrence of a heavy mast year for acorns and beech [9,22,23,24,25,26,27,28]. Damage is exacerbated near water sources and on wet soils [39,40,41,42] and decreases near human settlements [39,40]. Damage also intensifies during low-yield years resulting from insufficient precipitation [40].

Given these factors, the research tasks for this study were formulated to investigate the impact of the wild boar relative population density on the extent of damage to farmland areas, and to examine the relationship between the species composition of damaged agricultural crops and the species composition of all agricultural crops present in the farmland. This investigation tested two primary research hypotheses: Hypothesis 1 proposed that a decrease in relative population density, quantified by the size of the wild boar hunting bag, leads to a reduction in the damage level caused by these animals in farmlands. Hypothesis 2 was based on the premise that the percentage share of agricultural crops damaged by wild boar does not differ significantly from their proportional share across the entire area of the studied farmlands.

## 2. Materials and Methods

### 2.1. Study Area

**Figure 1:**
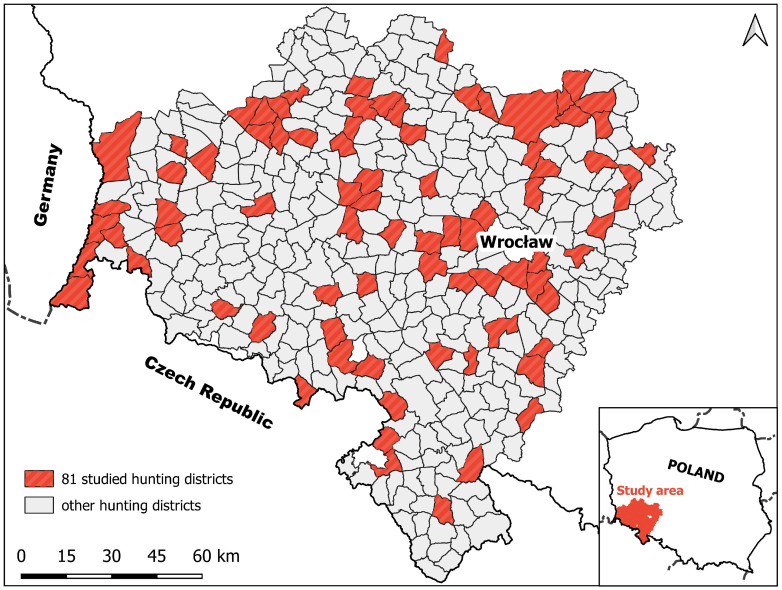
Location of hunting districts in south-western Poland.

The study was conducted in a forest-farmland in south-western Poland, partly adjoining the borders with Germany and the Czech Republic (Figure 1). The study area is divided into 360 hunting districts, which are administered by 264 hunting clubs and 16 state forest services. These hunting districts cover a total area of 16,581.1 km^2^ comprising 11,079.5 km^2^ of farmlands and 5506.6 km^2^ of forest. Game management is supervised by the Regional Directorate of State Forests in Wrocław [43].

According to GUS (2020) [44], various cereal crops constituted 0.58 of the total farmland area, whereas grasslands and rape seed constituted 0.07 and 0.1, respectively. The remaining part of the agricultural area (0.25) is occupied by maize (0.09), root crops (0.02) and other various crops (0.14).

The study area is strongly diverse in terms of natural resources, climate, and topography. The duration of the growing season varies from 150 days in the Sudety Mountains to 220 days in lowland habitats [45]. There are various degrees of fragmentation in both the lowland and upland forests, and there are also non-fragmented mountain forests in the Sudety Mountains. Coniferous forest types constitute 20% of the forested areas, whereas 61% is represented by mixed deciduous and mixed coniferous forest types, and the remaining portion (19%) by deciduous forest types. The main forest-forming species include pine (*Pinus sylvestris* L.), larch (*Larix decidua* Mill.), and spruce (*Picea abies* (L.) H.Karst) (a 73% share in tree stands), while deciduous tree species constitute 27%, with oak (*Quercus robur* L.) and beech (*Fagus sylvatica* L.) being of particular importance for wild boar in mast years [46].

Red deer (*Cervus elaphus*), fallow deer (*Dama dama*), roe deer (*Capreolus capreolus*), wild boar (*Sus scrofa*), and mouflons (*Ovis aries musimon*) have substantial economic significance for game management [47]. In 2016/17, the hunting season harvest expressed in thousands was 7.3 red deer, 17.7 roe deer, 0.4 fallow deer, 28.2 wild boar, and 0.5 mouflons.

Farmers very seldom use fencing agricultural crops. Using diversionary feeding by establishing in the forest so-called food strips is more common, but their impact upon the level of crops damaged by wild boar is not statistically significant [48].

### 2.2. Methods

In January 2017, a survey was sent to all hunting clubs concerning the characteristics of damage to agricultural crops caused by wild boar across 343 hunting districts. The survey covered data on wild boar-inflicted damage collected from March to February for three consecutive years, namely 2013/14, 2014/15, and 2015/16. Materials were received from 175 hunting districts (50.9% responses); over half of them (n = 94) showed no significant damage done by wild boar to farmland, or the data from the survey was incomplete. Complete wild boar damage information covering the crop species, surface area, date and number of damaged crops, compensation paid, and hunting bag data, as well as the total forest and farmland surface areas of the districts, was received from the remaining 81 districts. The total surface area of these districts amounted to 3491.2 km^2^, including 2449 km^2^ of farmland and 1042.2 km^2^ of forest. The total area includes fencing areas in the forest and in farmlands, but the built-up areas are not represented. Forest occupied 29.9% of the area hunting districts studied, and in hunting districts, ranged from 1.04% to 92.38%. The statistical analysis of wild boar-inflicted damage in each studied year was based on data from the same 81 hunting districts (mean area 43.1 km^2^).

Between the 2015/16 and 2022/23 period, as a result of African swine fever, the number of wild boars in the study area decreased. Therefore, in January 2024, hunting clubs that lease 81 hunting districts were recontacted in order to request data on wild boar harvest rates and the damage these animals had caused to agricultural crops. Data received from 50 of these hunting districts were compared with corresponding data acquired from the same 50 hunting districts in 2015/16.

The statistical analysis covers six categories of damaged crops: maize—*Zea mays*, various cereal crops (barley—*Hordeum vulgare*, wheat—*Triticum aestivum*, oat—*Avena sativa*, rye—*Secale cereale*), grasslands (meadows, pastures—*Molinio-Arrhenatheretea* R. Tx.), root crops (potato—*Solanum tuberosum*, sugar beets—*Beta vulgaris*, carrot—*Daucus* L.), rape seed—*Brassica napus*, and other crops (clover—*Trifolium* L., lupine—*Lupinus* L., strawberries—*Fragaria × ananassa* D., vetch—*Vicia* L.).

Distribution normality for the data on crop surface areas damaged by wild boar and the compensation paid in the subsequent 3 years (2013/14–2015/16) was verified using the Shapiro–Wilk test, and differences in the temporal variation in these variables were assessed using ANOVA with Friedman and Dunn’s post hoc tests. Due to the lack of a normal distribution of variables (Shapiro–Wilk test), statistical differences in relative population density, the percent of farmland areas damaged by wild boars, and the compensation value paid for destroyed crops between 2015/16 and 2022/23 were analyzed using the Mann–Whitney U test. The significance of agricultural crop selection by wild boars was tested using Bailey’s 95% simultaneous confidence intervals [49,50].

It was assumed that the relative population density index calculated from the number of wild boar harvested per km^2^ of a hunting district’s overall surface area was positively correlated with the population density of these animals [2,51,52]. The impact of relative population density index on the level of farmland damage caused by wild boar was investigated using nonlinear regression and Pearson’s correlation coefficient. The Statistica 13 software package was used in data processing.

## 3. Results

### 3.1. Temporal Variation Pattern of Crop Damage

The average percentage of farmland areas damaged by wild boar over 3 years (2013/14–2015/16) amounted to 0.86%. The damaged farmland area increased over three successive years, although a significant difference was noted only for grasslands and rapeseed (Table 1). The Shapiro–Wilk test showed a lack of normal distribution of data concerning the areas of agricultural crops damaged by wild boar and the value of compensation for losses in each of the studied years.

However, the area of wild boar damage to maize, various cereal crops, root crops, and various crops did not differ significantly between the years, as the *p*-values for the ANOVA Friedman test ranged 0.192 to 0.754. Significant differences between the years were shown for the areas of grasslands (*p* = 0.008) and rape seed (*p* = 0.048). Post hoc Dunn’s test confirmed the significant difference only for grasslands between years 2013/14 and 2015/16 (*p* = 0.011).

The values of compensation for wild boar damage paid to farmers increased throughout the three years (Table 1). However, compensation values for damaged maize, various cereal crops, root crops, and various crops did not differ significantly between the years because the *p* values for ANOVA Friedman test varied from 0.225 to 0.950. Significant differences between the years were shown for grasslands (*p* = 0.021) and rape seed (*p* = 0.022). Post hoc Dunn’s test confirmed a significant difference only for grasslands between the years 2013/14 and 2015/16 (*p* = 0.022).

The area of crops damaged by wild boar cumulated over the three years of the study (2098.2 ha) indicates that maize constituted 43.8% of the total damage area, while for various cereal crops and grasslands, the figures were 29.5% and 13.2%, respectively (Table 2). Farmers were compensated with EUR 884,500 for wild boar-inflicted damage. Percentage share of crops in damage compensation ranged from 43.8% (maize) to 1.5% (other crops). The average area of damage cases reached 0.95 hectares and varied from 0.24 hectares (root crops) to 1.49 hectares (maize). Converted per 1 hectare, the mean compensation paid to farmers was EUR 421.7, and the highest was for root crops (EUR 944.1), while the lowest was for grasslands (EUR 214.5) (Table 2).

### 3.2. Seasonal Variation Pattern of Crop Damage

**Figure 2:**
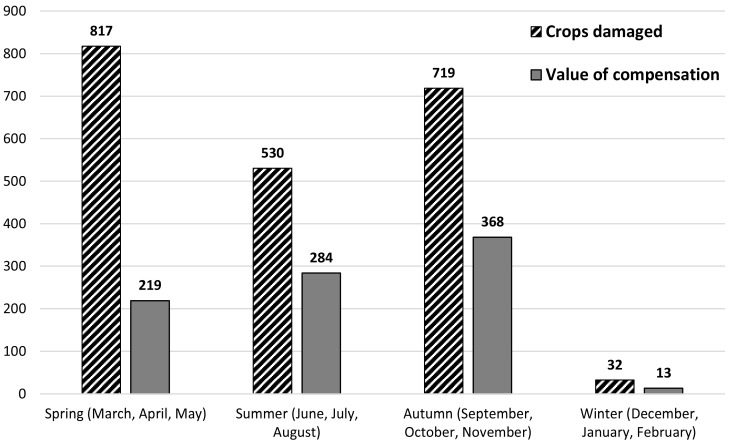
The seasonal distribution of crops damaged by wild boar (ha) and amount of compensation paid to farmers (EUR × 10^3^) over 3 years (2013/14–2015/16) in 81 hunting districts.

The cumulated area of damaged agricultural crops peaked in spring and autumn. This parameter was markedly lower in summer and lowest in winter. Damage compensation amounts increased from spring to summer and then from summer to autumn, and then finally decreased dramatically in winter (Figure 2). The total data for the three studied hunting seasons indicate that 73.2% of the damaged farmland area occurred in spring and autumn, while 74.0% of all compensation was attributed to summer and autumn. In winter, the share of both variables was lowest at 1.5% for each.

**Figure 3:**
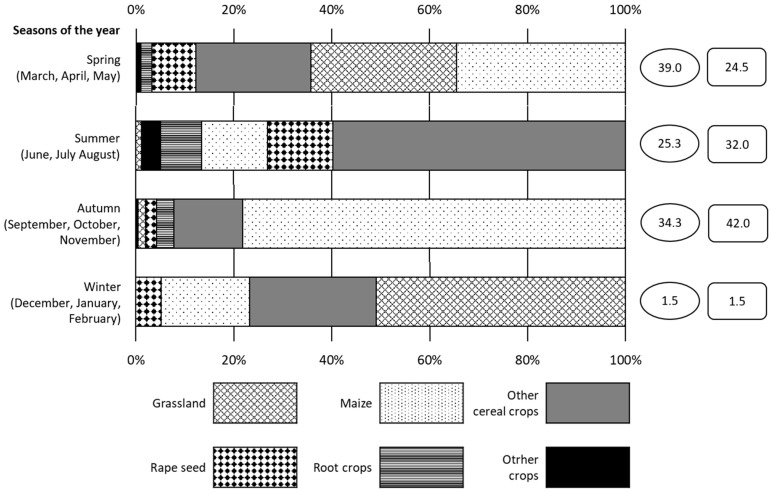
The share (%) of areas of each agricultural crop damaged by wild boar during the four seasons. Cumulated data is provided for three hunting seasons (2013/14–2015/16) covering 81 hunting districts. It is based on 2098.2 ha of crops damaged by wild boar and 884,500 EUR paid in compensation. The data in circles and rectangles present the percentage share of area damage and the percentage share of compensation in a given season, respectively, in relation to the area of all damage and to the total amount of compensation registered throughout the year.

Figure 3 presents three-year cumulated percentage areas of six agricultural crops damaged by wild boar during the four seasons of the year. In spring, the green foliage of maize as well as various cereal crops and grasslands amounted to 87.7% of all damaged crop areas. In summer, ripe cereal crops of various species constituted 59.7% of damaged areas. In turn, ripe maize strongly predominated among all crops damaged in autumn, representing 78.7% of the whole area of crop species destroyed by wild boar. In winter, more than half of the area of boar-inflicted damage (50.9%) was recorded in grasslands. A considerable percentage (25.8%) of damaged areas were covered by winter crops of various cereal species.

The largest year-round percentage of damaged crop areas, higher than the percentage of compensation paid (39.0% vs. 24.5%), occurred in spring. In summer and autumn, the percentage of compensation paid for the losses caused by wild boar was higher than the percentage of crop area damaged. Relative to spring, summer, and winter, autumn represents the highest percentage (42.0%) of compensation.

### 3.3. Crops Damaged vs. Availability of Crops

Differences between the availability of crops and their utilization by wild boars were assessed based on the proportion of crop areas available within the studied farmlands (see Section 2.1) and the proportion of crop areas damaged by these animals (see Table 2). The proportion of area utilized by wild boars for maize, grasslands, and root crops was higher than the proportion of their availability (A). A crop selection index (U/A) greater than unity indicates that these three crop types were preferred by wild boars. The proportions of availability for these species fell below the lower bound of the confidence intervals; therefore, their utilization significantly differed from their availability (Table 3). Conversely, the proportion of area utilized by wild boars for various cereal crops, rapeseed, and other crops was lower than the proportion of their availability (U < A). A crop selection index (U/A) lower than unity suggests that these crop species were not preferred by wild boars. The proportions of availability for these species fell above the upper bound of the confidence intervals, and consequently, their utilization significantly differed from their availability (Table 3).

In conclusion, the research hypothesis concerning no significant differences between the proportion of damaged crop areas and the proportion of available crop areas was negatively verified. The utilization of agricultural crops by wild boars did not exhibit random use, as the proportions of available crop areas did not fall within the confidence interval limits.

### 3.4. Population Harvest Rate and Crop Damage

The increase in area of damaged crops in 3 successive years was proportional to the wild boar harvest rate. The hunting bag of this animal in the study area increased from 1.53 individual/km^2^ in 2013/14 to 1.83 and 2.30 animals/km^2^ in the following two years, respectively. Throughout the three study years, the area of crop damage grew from 641.6 ha in the year 2013/14 to 790.2 ha in 2015/16 (Table 1).

**Figure 4:**
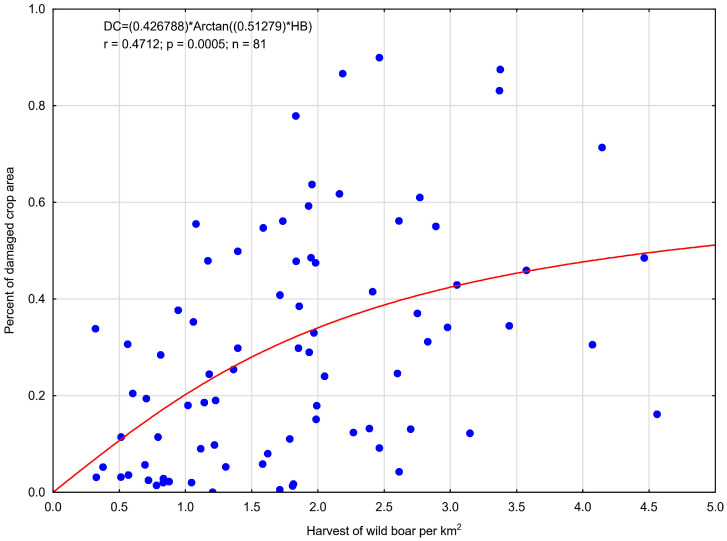
Relationship between the percent of damaged crop area (DC) and the harvest rate of wild boar (HB) based on 81 hunting district data in hunting season 2014/15.

Calculations performed for 81 hunting districts showed a positive correlation significant between the harvest rate of wild boar and the percentage of farmland area damaged by these animals only for the 2014/15 hunting season (Figure 4). The following equation presents the demonstrated relationship:

DC = (0.426788) × Arctan ((0.51279) × HB); r = 0.4712; *p* = 0.0005; n = 81

where DC denotes the percentage of farmland area damaged by wild boar and HB denotes the wild boar harvest rate (N/km^2^). The coefficient of determination (r^2^) indicates that the harvest rate determined 22.2% of damaged farmland areas.

The above relationship was not significant for data representing the 2013/14 hunting season (r = 0.424; *p* = 0.411) and 2015/16 hunting season (r = 0.501; *p* = 0.098).

**Figure 5:**
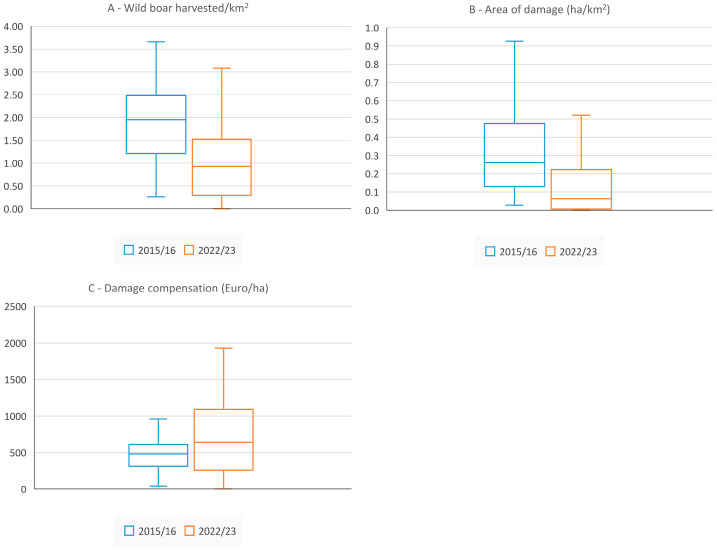
Comparison of the average values (median) of variables (**A**) Wild boar harvested/km^2^, (**B**) Area of damage (ha/km^2^), and (**C**) Damage compensation (€/ha) between the 2015/2016 and 2022/2023 seasons.

A significant link between the wild boar harvest rate and crop damage was demonstrated for 50 hunting districts (Table 4). Figure 5 presents a comparison of the average values (median) of variables: (A) Wild boar harvested/km^2^, (B) Area of damage (ha/km^2^), and (C) Damage compensation (€/ha) between the 2015/2016 and 2022/2023 seasons. The results of the Mann–Whitney test confirmed significant differences for variables A and B (the *p*-values were 0.000004 and 0.000013, respectively). However, the difference in the average damage compensation (€/ha) was not statistically significant (*p* = 0.0945).

## 4. Discussion

### 4.1. Seasonal Variation in Damage Occurrence

The seasonal pattern of agricultural crop damage by wild boar (Figure 2) is determined by seasonal changes in energy requirements of the population [53,54,55] and by the standing crop and quality of the potential food base that farmlands and forest habitats represent for wild boar.

The high level of damage in spring is associated with the farrowing period, as most piglets are born in March and April [6,56]. To feed piglets, the daily energy budget of lactating females doubles, as does their food intake [57]. In late spring, sows begin the process of restoring their energy reserves that have been spent on lactation, and piglets start to put on weight by obtaining food on their own [58]. Other individuals foraging to recover winter losses in body mass also contribute to the high level of damage [59].

In May, young growth stages of maize and various cereal crops present a highly digestible wild boar food [8]. Thawed soil in grasslands enables earthworms and grubs to remigrate towards the upper soil layers [60] and makes it possible for wild boar to access food via rooting. The standing crop of earthworms and grubs may amount to 77 g and 34 g/m^2^, respectively [61]. These food resources have a much higher level of metabolic energy than plant forage [8]. This is particularly true of grubs, with fat reserves constituting approx. 45% of their body mass [62]. Food obtained by wild boar via rooting also includes underground parts of grasses and herbs. Given the energy reserves directed there for winter, these plant parts have a high nutritive value. The standing crop of underground plant parts in meadows and pastures exceeds the biomass of the underground parts of ground flora plants in forests several times [63,64]. However, the energy costs of rooting reduce the amount of metabolic energy that wild boar put into reproduction, growth, and body mass recovery.

A considerable summertime reduction in the surface area of farmlands damaged by wild boar (Figure 2) was also demonstrated in central and east-central Poland [65,66]. This was likely caused by the growth of potential highly nutritious food base for wild boar in forest areas. During the mentioned period, forests offer these animals a high standing crop of ground flora plants and soft mast production [67,68], as well as substantial amounts of browse [69] and invertebrates in the soil and on its surface [70,71]. The consumption of summer food base in forests and the mature seeds of various cereal species in fields by wild boar (Figure 2) considerably affects body mass gain in piglets and yearlings. The results of this study confirm the findings of Bieber and Ruff [72], that wild boar are adapted to the use of diverse plant and animal food sources and they can swiftly alternate between them according to availability.

The attractiveness of farmlands for wild boar increases in autumn. This is due to maize cultivation, with its calorie-dense seed production reaching a level of approx. 7.29 tons/ha (GUS 2024) [38]. This provides wild boar with easier fat reserve accumulation before winter, and males need fat reserves for the mating season.

In winter, farmlands present no particular significance as a potential food base for wild boar. Crop biomass is very low, while earthworms and grubs descend to the lower unfrozen soil layers. The frozen soil prevents wild boar from rooting and utilizing the underground parts of grasses and herbs in meadows and pastures. In winter, wild boar are forced to feed mainly on forest food, such as ground flora plants and browse [58], which does not cover their daily energy budget in non-mast years for oak and beech. This is when the animals utilize their protein and fat reserves and lose body mass [73].

### 4.2. Management Implications

Calculations made for northeastern Poland indicate that reducing the population density of wild boar from 7.9 to 5.0 animals/km^2^ leads to a 38% decrease in the area damaged [74]. This is also confirmed by the relationship found in the presented study. Similar results on lowered damage to agricultural crops as an effect of reducing wild boar population densities were obtained in Switzerland [75] and the USA [76]. The authors of numerous papers suggest that the best method to mitigate damage caused by wild boar to farmlands is to regulate the population densities of these animals [24,26,77]. Therefore, reducing the population density seems to be a universal and effective method to limit the damage by wild boars to farmlands.

## 5. Conclusions

Although the percentage area of farmlands damaged by wild boar in 81 hunting districts (mean = 0.86%, see Section 3) does not pose a substantial economic problem at the regional scale, it can significantly reduce crop yields at the level of several-hectare agricultural farms and specific damage cases. Additionally, wild boar-inflicted losses cause high emotional costs among local farmers associated with wasted efforts in their crop production work [27,78,79,80].

Reducing the population density will significantly lower the damage inflicted by wild boar on agricultural crops as long as the reduction is carried out across the local range of the species throughout the year. However, such population density control is only possible if wild boar management is based on a reliable population size assessment and its net annual population increase.

## Figures and Tables

**Table 1:** Agricultural crops damaged by wild boar and damage compensation in 81 hunting districts over 3 years. The compensation values were calculated on the basis of the market value of damaged crops expressed in EUR.

Crop Type	Years 2013/2014			Years 2014/2015			Years 2015/2016		
	Area of Damage (ha)	Damage Cases (N)	Damage Compensation (EUR × 10^3^)	Area of Damage (ha)	Damage Cases (N)	Damage Compensation (EUR × 10^3^)	Area of Damage (ha)	Damage Cases (N)	Damage Compensation (EUR × 10^3^)
Maize	326.5	210	147.6	269.1	201	109.7	325.3	208	171.5
Other cereal crops	182.2	272	61.1	200.9	226	60.1	235.1	251	78.7
Grassland	61.9	76	13.6	97.7	114	22.2	116.8	128	23.5
Rape seed	28.0	30	19.2	58.9	50	40.7	76.1	48	40.1
Root crops	30.5	131	29.2	30.3	137	30.3	27.0	102	23.3
Other crops	12.5	10	6.7	9.4	12	4.3	10.0	10	3.0
Total	641.6	729	277.4	666.3	740	267.3	790.3	747	340.1

**Table 2:** Characteristics of crops damaged by wild boar in the study area over a 3-year period (2013/14–2015/16). Compensation values were calculated on the basis of the market value of damaged crops expressed in EUR. Data were collected in 81 hunting districts.

Crop Type	Area of Crops Damaged (ha)	Percentage Share of Area Damaged Crops	Damage Compensation (EUR × 10^3^)	Percentage Share of Crops in Damage Compensation	Number of Damage Cases	Mean Area of One Damage Case (ha)	Damage Compensation (EUR per 1 ha)
Maize	920.9	43.8	428.8	48.5	619	1.49	465.6
Other cereal crops	618.2	29.5	199.9	22.6	749	0.83	323.3
Grassland	276.5	13.2	59.3	6.7	318	0.87	214.5
Rape seed	163.0	7.8	100.0	11.3	128	1.27	613.5
Root crops	87.7	4.2	82.8	9.3	370	0.24	944.1
Other crops	31.9	1.5	14.0	1.6	32	1.00	438.9
Total—weighted mean	2098.2	100.0	884.8	100.0	2216	0.95	421.7

**Table 3:** Selection of various agricultural crops by wild boars estimated by Bailey’s simultaneous intervals based upon data from south-western Poland. Crops preferred (+) and not preferred (−) by wild boar.

Crop Type	Area of Crops (ha)	Proportions Areas of Crops Available (A)	Area of Crops Damaged (ha)	Proportions Areas of Crops Damaged (U)	Crop Selection Index (U/A)	Bailey’s 95% Simultaneous Confidence Intervals	Crop Selection
Maize	22,041	0.09	921	0.44	4.88	0.4101–0.4677	(+)
Other cereal crops	142,042	0.58	618	0.29	0.51	0.2684–0.3213	(−)
Grassland	17,143	0.07	276	0.13	1.88	0.1125–0.1519	(+)
Rape seed	24,490	0.10	163	0.08	0.78	0.0629–0.0942	(−)
Root crops	4,898	0.02	88	0.04	2.10	0.0311–0.0547	(+)
Other crops	34,286	0.14	32	0.02	0.11	0.0089–0.0236	(−)
Total	244,900	1.00	2098	1.00	---	---	---

**Table 4:** Characteristics of crops damaged by wild boar in the study area over two years. The compensation values were calculated on the basis of the market value of damaged crops expressed in EUR. Data for each year were collected in 50 hunting districts. I—area of damage (ha), II—percentage share of area of damage crops (%), III—total amount of damage compensation (EUR × 10^3^), IV—percentage share of crops in damage compensation (%), and V—damage compensation (EUR per 1 hectare) *—weighted mean.

Crop Type/Season	I		II		III		IV		V	
	2015/16	2022/23	2015/16	2022/23	2015/16	2022/23	2015/16	2022/23	2015/16	2022/23
Maize	184.4	119.2	38.2	46.5	105.6	124.1	50.1	56.8	572.9	1041.4
Other cereal crops	159.9	48.2	33.1	18.8	54.1	38.3	25.6	17.5	338.5	796.3
Grassland	75.6	46.9	15.7	18.4	14.7	21.1	7.0	9.7	194.7	450.1
Rape seed	45.5	32.5	9.4	12.7	19.6	21.3	9.3	9.7	431.1	654.9
Root crops	14.9	3.4	3.1	1.3	16.0	9.3	7.6	4.3	1071.8	2700.2
Other crops	2.4	6.0	0.5	2.3	0.9	4.3	0.4	2.0	364.6	728.6
Total/weighted mean *	482.7	256.2	100.0	100.0	211.0	218.5	100.0	100.0	437.1 *	852.8 *

## Data Availability

The data that support the findings of this study are available from the corresponding author upon reasonable request.
